# Notice—Last Call

**Published:** 1877-12-20

**Authors:** 


### NOTICE—LAST CALL.

The Red Cross means that your subscription for 1877 has not been paid. Please settle at once.

